# A TFEB‐lysosomal pH signaling pathway in tauopathy

**DOI:** 10.1002/alz70861_108383

**Published:** 2025-12-23

**Authors:** Hui Zheng

**Affiliations:** ^1^ Baylor College of Medicine, Houston, TX USA

## Abstract

**Background:**

Lysosomes play a critical in the clearance of macromolecules such as protein aggregates. The transcription factor TFEB is a master regulator of lysosomal function. TFEB responds to lysosomal stress and translocates to nucleus where it mediates gene expression. TFEB targets include subunits of the v‐ATPase essential for lysosomal acidification. Here we investigated the role of TFEB‐v‐ATPase signaling in tau pathology.

**Method:**

We created mutant mice in which the TFEB‐v‐ATPase signaling pathway is disrupted, and further crossed it with tau transgenic mice. We performed single nucleus RNA‐sequencing (snRNA‐seq) to examine the gene expression changes in various cell types. In addition, we performed immunostaining and biochemical analysis to characterize tau pathology and glial cell morphologies.

**Result:**

We found that disruption of the TFEB‐v‐ATPase signaling leads to increased tau pathology, validating an important role of the lysosome in tau degradation. Surprisingly, we found that microglia can no longer be activated in the lysosomal mutants in response to tau pathology. snRNA‐seq showed that microglia activation is associated with upregulation of both immune and lysosomal pathway genes. Reduced lysosomal TFEB‐v‐ATPase signaling leads to the downregulation of the immune pathways, demonstrating a critical role of the lysosome in microglia activation.

**Conclusion:**

Our studies identify a novel lysosome‐immune relationship in tauopathy and Alzheimer's disease.

